# Encouraging productive struggle in the age of generative AI

**DOI:** 10.1016/j.patter.2026.101540

**Published:** 2026-05-08

**Authors:** Andrei Biswas, Rajagopal Venkatesaramani

**Affiliations:** 1 Khoury College of Computer Sciences, Northeastern University, Boston, MA 02115, USA

## Abstract

Computer science (CS) educators are increasingly concerned about students offloading thinking to generative AI (GenAI) tools. Reflecting on experiences teaching a foundational AI course, the authors argue that the community must rethink supportive environments for productive failure and better connect CS concepts to industry contexts to encourage interdisciplinary thinking alongside technical depth.

## Main text

Over the last half a century, nearly every educator has experienced at least one event that majorly impacted the pedagogical landscape. The introduction of digital calculators and the internet drew widespread concern (including protests) among educators, and today, the same can be said of generative AI (GenAI) tools. We believe that computer science (CS) fundamentally concerns itself with computational problem-solving rather than mere coding proficiency. But if that is the premise, then why would students cheat themselves of the very exercise that they signed up for to become CS practitioners? Our position is that GenAI tools have exacerbated an existing problem: a student pursuing a CS degree in today’s technological landscape lacks sufficient motivation to engage in productive struggle^1^ in a traditional direct-instruction setting and, moreover, faces active disincentives to do so.

The first such disincentive, in our view, is students’ perceived importance of a perfect grade point average (GPA). We attribute such perceptions to (1) expectations set by upward trends in average high school GPA, (2) the consequences of high variance in letter grade assignments across courses, instructors, and institutions, and (3) artificial grade inflation.^2^ Although demonstrated effort may contribute to a student’s grade at the middle and high school level, this is seldom the case in higher learning institutions, which emphasize outcomes. For a student who is used to being rewarded for working hard, being asked to spend hours on a problem where mistakes can cost them their grade can be a frustrating experience. Despite this, students are quick to acknowledge that effort is inherently subjective and cannot be graded using a quantitative rubric. Such situations can lead to low morale, which in turn may lead students to seek the path of least resistance to their desired letter grade. In addition to our own experiences, prior research^3^ also indicates that students who score lower grades in initial courses refrain from taking more advanced courses, presumably due to low morale or to boost their overall GPA, or both, and yet end up with a lower cumulative GPA on average. Point (2) is challenging to address, since academic freedom and standardization can be viewed as somewhat incompatible. Our position is that academic freedom is central to scientific and pedagogical innovation, and therefore, must be preserved. However, we would like to acknowledge the challenges this presents for CS education. Grade-oriented students have a tendency to seek out sections or courses with higher average grade outcomes—whatever the reasons for such outcomes may be—even when the pedagogical techniques employed by instructors perceived as more “difficult” are better aligned with their needs. We also note that we do not fault such students for being grade oriented; rather, we wish to highlight the comedy of errors that has led to higher grades being perceived as the sole indicator of better post-graduation outcomes. Finally, grade inflation is a well-studied problem in higher education, which subsequently sets unrealistic standards and expectations for graduates. This is often a byproduct of adjunct and teaching-track faculty contracts and merit reviews being conditioned on student evaluations of teaching (SET), which have been shown to be notoriously unreliable indicators of teaching effectiveness.^4^ These factors place undue pressure on faculty to placate students through diluted grading standards as a safeguard against loss of employment, and it is our opinion that faculty (adjunct/part-time/teaching-track faculty in particular) often feel inadequately supported in their defense of academic rigor.

The second key disincentive is the ease of access and short response times that GenAI tools offer, combined with widespread adoption. Superficially, those qualities seem like a point in favor of AI tools. But such rapid response times can be counterproductive to the objective of exercises designed to help students build the ability to formulate and express, rather than to simply arrive at and report solutions. In our experience, students are increasingly reluctant to engage with time-consuming problem-solving exercises unless explicitly encouraged to do so in a supportive environment such as office hours. We see this as an opportunity and a signal that we need to reflect as educators and adapt our pedagogy accordingly. We are further concerned that the need for such solutions arises due to a more fundamental problem at institutions of higher learning—in that declining faculty recruitment and retention rates, higher teaching loads, enrollment growth, and challenges with scalable teaching assistant (TA) training and support have made it increasingly difficult for courses to offer sufficient and timely support to every student. While active learning strategies have proven effective, the scale of large classes can be a deterrent to an instructor effectively deploying them. Larger classes also make the student experience notably worse and have been shown to be less effective in building computational thinking skills. Recommendations from both the National Academies^5^ and the Computing Research Association^6^ mirror our concerns and highlight the need for institutions to respond by ensuring reasonable student-faculty ratios, competitive teaching loads and salaries for faculty, and investment in improvements to graduate education and robust TA training programs.

The third key disincentive is that GenAI tools produce plausible-sounding, human-like text. This capability leads many to perceive these tools not as fallible technologies but as sources of authoritative knowledge. Media coverage of GenAI tools is dominated by sensationalism and uncritical reporting, with news outlets often presenting these technologies as “capable of solving nearly any problem” through “largely positive evaluations and economic framing.”^7^ This combination becomes particularly problematic given that large language models (LLMs) frequently generate false information, which can mislead students. While professionals demonstrate increased skepticism and fact-checking behavior when AI authorship is explicitly disclosed, students engaged in learning tasks may not maintain such critical evaluation, especially given the overwhelmingly positive media narratives. As institutions expand GenAI tool access to students, they must establish institutional policies that preserve academic freedom and integrity while enabling students to use these tools for meaningful learning.

To address the challenge of grade-oriented thinking, we have found it effective to frame lectures as a progression of self-discovery steps rather than offering information upfront and testing comprehension afterward. Instead of the traditional format, the instructor continually poses leading questions until the class collectively arrives at key takeaways in incremental steps. This scalable active learning strategy addresses all categories of Bloom’s Taxonomy while building students’ confidence to speak up and engage with problems directly. Crucially, the instructor demonstrates deductive reasoning by treating incorrect or partially correct answers not as failures, but as steps toward finding the right answer. Student feedback credits this approach with fostering a sense of discovery, enjoyment, and achievement throughout the learning process, and cites it as a motivator for increased in-person office hours attendance.

We also recognize the importance of enabling an environment where students feel comfortable adapting to tools that industry uses, which requires plainly differentiating between constructive and problematic AI use. To this end, we divide homework into theoretical “problem sets” and programming assignments. For theoretical work, we limit AI assistance to linguistic refinement and require students to submit both their original unedited answers as well as the AI-enhanced versions, helping graders verify the authenticity of their conceptual understanding. For programming assignments, we permit broader AI usage but with mandatory attribution through code comments. Most critically, we enforce a comprehension requirement: students must be able to explain any AI-generated code when seeking TA support or filing regrade requests, ensuring that AI amplifies rather than replaces learning.

Increased in-person office hours attendance necessitates rethinking how we deliver TA support. Student TAs are “near-peers,” many of whom completed the same courses just one semester prior. This fresh perspective on course challenges and recent experience with the same conceptual hurdles makes them relatable to students and transforms office hours into collaborative learning experiences. TAs provide support that transcends technical problem-solving: they create spaces where students can safely explore ideas, express confusion, and debate concepts. TAs are also well positioned to connect the material to how they applied these concepts during internships or jobs, providing additional motivation for students to pursue productive struggle. The most transformative element of our approach involves implementing pair programming during office hours. Research shows that pair programming improves both project performance and course completion rates while reducing frustration and increasing confidence through peer connections. Pair programming sessions during TA office hours create a scalable model that fosters collaborative problem-solving and the ability to scope problems for human explanation, both of which are technical soft skills sought within the industry. Training TAs to serve as communication bridges between students and instructors creates a dynamic loop of continuous improvement. This real-time insight into student misconceptions allows instructors to adapt their teaching accordingly, ensuring that support evolves with emerging needs.

Finally, we turn our attention to aligning CS pedagogy with industry practices and expectations. Within the past decade, the number of students seeking double majors has risen sharply, especially in pairings such as business and CS. According to NCES data, the share of college graduates earning more than one credential doubled from 6% to 12% between 2014 and 2024. The economic rationale is substantial: recent research^8^ shows that double majors reduce income shock by 56%, with unrelated combinations (such as engineering with business) providing nearly double the protection of related pairs: 64% vs. 36%. These students are not only more financially resilient but better positioned to leverage their interdisciplinary expertise. Industry signals a similar shift toward analytical thinking, resilience, and creative thinking. Companies like Meta now allow AI tool usage during technical interviews, evaluating not whether candidates can write algorithms from memory but how they reason about, communicate, and iterate on code produced with AI assistance. Interviewers observe how candidates scope problems, explain their thought process, and verify AI-generated output; these are significantly more complex skills to demonstrate than prompting a way to a solution. We believe that students who engage in productive struggle are much better positioned to excel in such interview settings because they possess a deep understanding of core concepts.

The integration of GenAI into computing is irreversible, but what makes a strong CS graduate has not fundamentally changed: the ability to formulate and algorithmically solve complex problems. While GenAI makes it easier for students to sidestep the very struggle that builds this skill, our experience has shown that aligning student attitudes to what benefits them, and what the industry ultimately expects, is achievable. Self-discovery-driven lectures build the necessary confidence needed to work through difficult problems. Near-peer TAs and pair programming create environments where struggle is collaborative rather than isolating. Connecting CS to the broader landscape gives students a reason to engage deeply with the material beyond the next test. The goal is not to shield students from AI tools, but to ensure they experience productive struggle in controlled environments to build genuine understanding before leveraging these tools for amplification. Our enduring responsibility as educators is to ensure that students discover joy in challenge, wisdom in failure, and confidence in their capacity to think across boundaries.

## Declaration of interests

The authors declare no competing interests.
